# Supplementary material for: Virtual Biopsy for Diagnosis of Chemotherapy-Associated Liver Injuries and Steatohepatitis: A Combined Radiomic and Clinical Model in Patients with Colorectal Liver Metastases
Source: Cancers (Basel). 2021 Jun 20;13(12):3077. doi: 10.3390/cancers13123077 (PMC8234168; doi:10.3390/cancers13123077)
Supplement: Supplementary file 1 [file cancers-13-03077-s001.zip › cancers-1164950-supplementary.pdf]

Supplementary Materials

Virtual Biopsy for Diagnosis of Chemotherapy-Associated Liver Injuries and Steatohepatitis: A Combined Radiomic and Clinical Model in Patients with Colorectal Liver Metastases

Guido Costa, Lara Cavinato, Chiara Masci, Francesco Fiz, Martina Sollini, Letterio Salvatore Politi, Arturo Chiti, Luca Balzarini, Alessio Aghemo, Luca di Tommaso, Francesca Ieva, Guido Torzilli and Luca Viganò

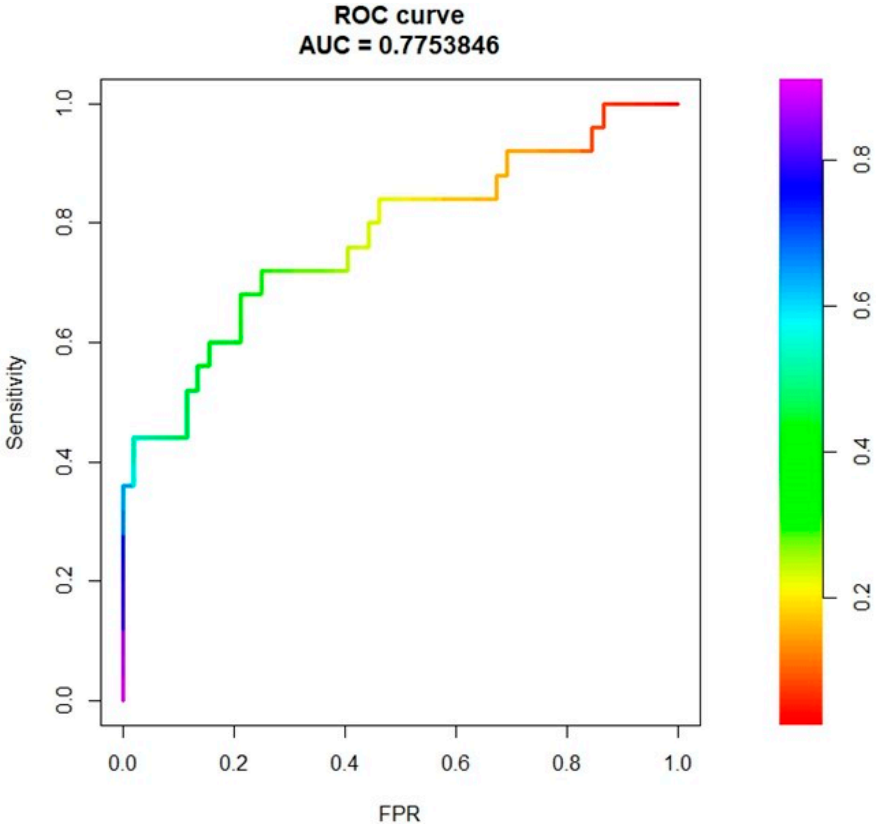

Figure S1. ROC curve analysis of the clinical and laboratory model for the prediction of grade 2–3 sinusoidal dilatation.

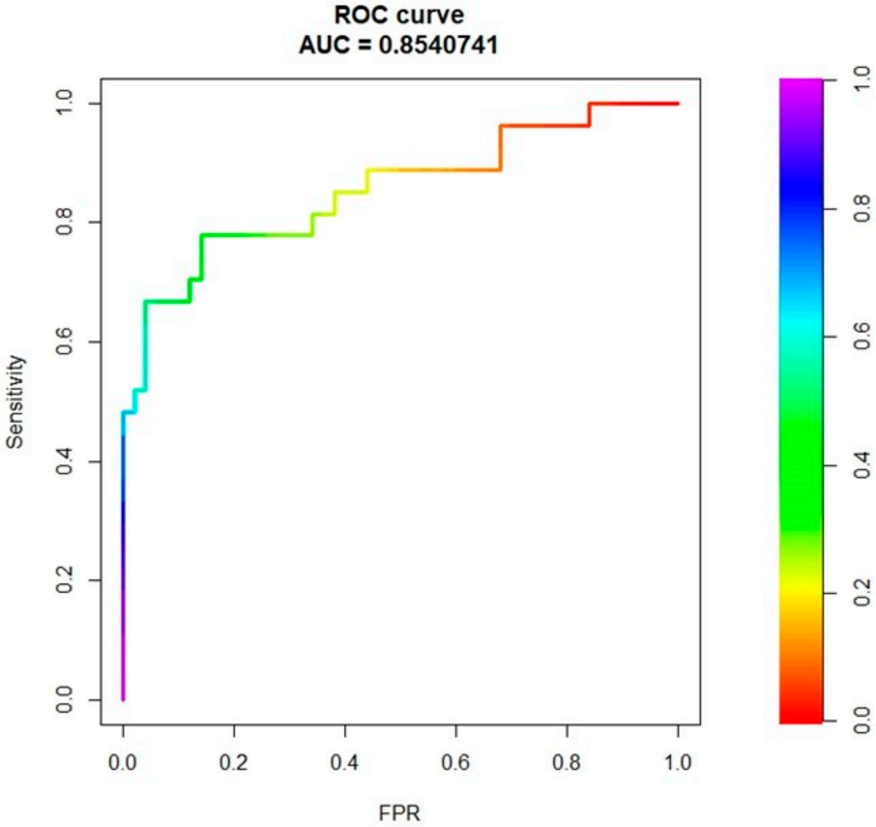

Figure S2. ROC curve analysis of the clinical and laboratory model for the prediction of NRH.

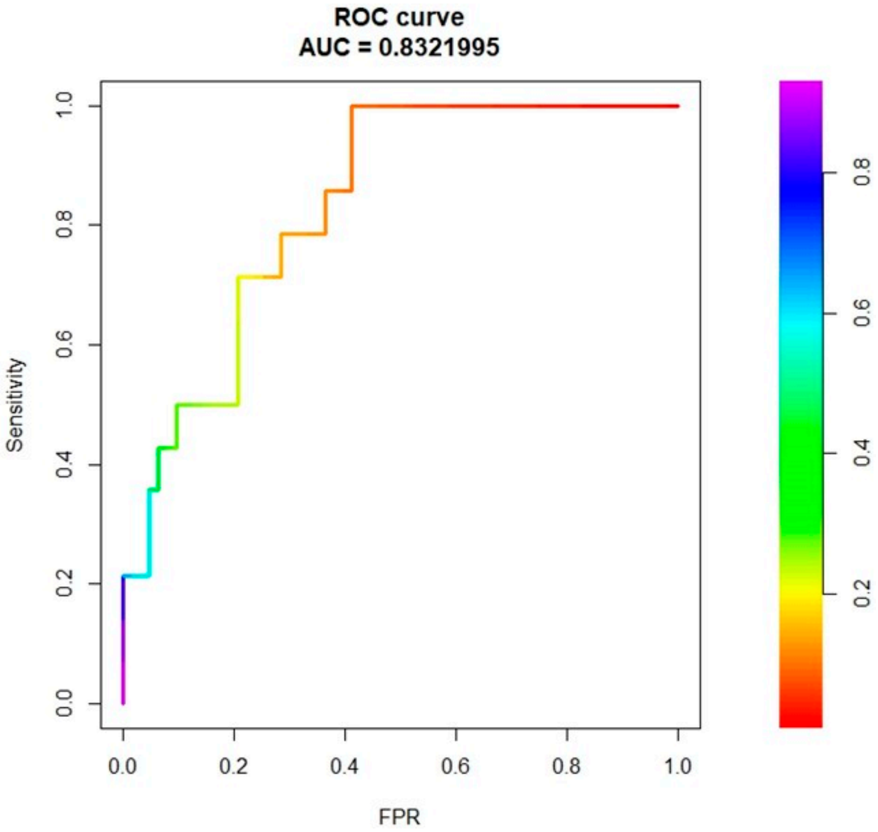

Figure S3. ROC curve analysis of the clinical and laboratory model for the prediction of NASH

Table S1. Chemotherapy-related liver injuries.

**Sinusoidal Dilatation**, classified according to Rubbia-Brandt et al. [1]	
**Grade 1**	Centrilobular involvement limited to one-third of the lobular surface
Grade 2	Centrilobular involvement extending in two-thirds of the lobular surface
Grade 3	Complete lobular involvement
**Nodular regenerative hyperplasia**, classified according to Wanless et al. [2]	
Grade 1	Focal occasionally distinct nodular hyperplasia on reticulin staining but indistinct on hematoxylin and eosin staining
Grade 2	Focal distinct nodular hyperplasia apparent on hematoxylin and eosin staining, clearly highlighted on reticulin staining
Grade 3	Diffuse nodular hyperplasia, distinct in most areas on hematoxylin and eosin staining and highlighted on reticulin staining
**Steatohepatitis**, components classified according to Kleiner et al. [3]<br>Definition according to EASL guidelines [4], i.e., the joint presence of steatosis, ballooning and lobular inflammation.	
Steatosis	
Grade 1	<30% of involved hepatocytes
Grade 2	30–60% of involved hepatocytes
Grade 3	>60% of involved hepatocytes
Lobular inflammation	
Grade 1	<2 foci per 200x field
Grade 2	2–4 foci per 200x field
Grade 3	> 4 foci per 200x field
Hepatocellular ballooning	
Grade 1	Few balloon cells
Grade 2	Many cells/prominent ballooning

Table S2. Association between CALI and operative outcomes.

**Grade 2–3 Sinusoidal Dilatation**			
	**N (*n* = 53)**	**Y (*n* = 25)**	***p***
90-day mortality	- (0%)	- (0%)	-
Major morbidity (grade ≥III) *	4 (7.6%)	1 (4.0%)	1.000
Grade B-C liver failure **	1 (1.9%)	1 (4.0%)	0.541
**NRH**			
	**N (*n* = 51)**	**Y (*n* = 27)**	***p***
90-day mortality	- (0%)	- (0%)	-
Major morbidity (grade ≥III) *	4 (7.8%)	1 (3.7%)	0.654
Grade B-C liver failure **	1 (2.0%)	1 (3.7%)	1.000
**Steatohepatitis**			
	**N (*n* = 64)**	**Y (*n* = 14)**	***p***
90-day mortality	- (0%)	- (0%)	-
Major morbidity (grade ≥III) *	3 (4.7%)	2 (14.3%)	0.281
Grade B-C liver failure **	1 (1.6%)	1 (7.1%)	0.329

* According Clavien-Dindo classification, please see Dindo et al., Ann Surg 2004 [5]; ** according to ISGLS classification, please see Rahbari et al., Surgery 2011 [6].

References

1 Rubbia-Brandt, L.; Audard, V.; Sartoretti, P.; Roth, A.D.; Brezault, C.; Le Charpentier, M.; Dousset, B.; Morel, P.; Soubrane, O.; Chaussade, S.; et al. Severe hepatic sinusoidal obstruction associated with oxaliplatin-based chemotherapy in patients with metastatic colorectal cancer. *Ann. Oncol*. **2004**, *15*, 460–466, doi:10.1093/annonc/mdh095.

2 Wanless, I.R. Micronodular transformation (nodular regenerative hyperplasia) of the liver: A report of 64 cases among 2500 autopsies and a new classification of benign hepatocellular nodules. *Hepatology* **1990**, *11*, 787–797, doi:10.1002/hep.1840110512.

3 Kleiner, D.E.; Brunt, E.M.; Van Natta, M.; Behling, C.; Contos, M.J.; Cummings, O.W.; Ferrell, L.D.; Liu, Y.C.; Torbenson, M.S.; Unalp-Arida, A.; et al. Design and validation of a histological scoring system for nonalcoholic fatty liver disease. *Hepatology* **2005**, *41*, 1313–1321, doi:10.1002/hep.20701.

4 Marchesini, G.; Day, C.P.; Dufour, J.F.; Canbay, A.; Nobili, V.; Ratziu, V.; Tilg, H.; Roden, M.; Gastaldelli, A.; Yki-Jarvinen, H.; et al. EASL-EASD-EASO Clinical Practice Guidelines for the management of non-alcoholic fatty liver disease. *J. Hepatol.* **2016**, *64*, 1388–1402, doi:10.1016/j.jhep.2015.11.004.

5 Dindo, D.; Demartines, N.; Clavien, P.A. Classification of surgical complications: a new proposal with evaluation in a cohort of 6336 patients and results of a survey. *Ann Surg.* **2004**, *240*, 205–213. doi: 10.1097/01.sla.0000133083.54934.ae.

6 Rahbari, N.N.; Garden, O.J.; Padbury, R.; Brooke-Smith, M.; Crawford, M.; Adam, R.; Koch, M.; Makuuchi, M.; Dematteo, R.P.; Christophi, C.; et al. Posthepatectomy liver failure: a definition and grading by the International Study Group of Liver Surgery (ISGLS). *Surgery* **2011**, *149*, 713–724. doi: 10.1016/j.surg.2010.10.001.
